# Recent Developments in Linear Interaction Energy Based Binding Free Energy Calculations

**DOI:** 10.3389/fmolb.2020.00114

**Published:** 2020-06-17

**Authors:** Eko Aditya Rifai, Marc van Dijk, Daan P. Geerke

**Affiliations:** AIMMS Division of Molecular and Computational Toxicology, Department of Chemistry and Pharmaceutical Sciences, Vrije Universiteit Amsterdam, Amsterdam, Netherlands

**Keywords:** binding affinity computation, free energy calculation, molecular simulation, linear interaction energy, protein flexibility, binding promiscuity, applicability domain, reliability estimation

## Abstract

The linear interaction energy (LIE) approach is an end–point method to compute binding affinities. As such it combines explicit conformational sampling (of the protein-bound and unbound-ligand states) with efficiency in calculating values for the protein-ligand binding free energy Δ*G*_*bind*_. This perspective summarizes our recent efforts to use molecular simulation and empirically calibrated LIE models for accurate and efficient calculation of Δ*G*_*bind*_ for diverse sets of compounds binding to flexible proteins (e.g., Cytochrome P450s and other proteins of direct pharmaceutical or biochemical interest). Such proteins pose challenges on Δ*G*_*bind*_ computation, which we tackle using a previously introduced statistically weighted LIE scheme. Because calibrated LIE models require empirical fitting of scaling parameters, they need to be accompanied with an applicability domain (AD) definition to provide a measure of confidence for predictions for arbitrary query compounds within a reference frame defined by a collective chemical and interaction space. To enable AD assessment of LIE predictions (or other protein-structure and -dynamic based Δ*G*_*bind*_ calculations) we recently introduced strategies for AD assignment of LIE models, based on simulation and training data only. These strategies are reviewed here as well, together with available tools to facilitate and/or automate LIE computation (including software for combined statistically-weighted LIE calculations and AD assessment).

## 1. End-Point Methods and Linear Interaction Energy

Mutual molecular recognition is the starting point for a wide variety of biological processes (Gohlke and Klebe, 2002). Binding affinity governs ligand binding to target proteins, and being able to quantitatively understand and predict affinity in terms of binding free energy (Δ*G*_*bind*_) can greatly support lead finding and/or optimization in the drug discovery process (Pohorille et al., 2010). Hence, improved efficiency and accuracy of computer–aided protein–ligand affinity methods play a pivotal role in accelerating and increasing success rates of drug discovery and design. Δ*G*_*bind*_ computation is still challenging, considering that virtual screening based on docking and scoring typically lacks sufficient accuracy, whereas use of rigorous alchemical methods can be too compute intensive for high–throughput scenarios, especially in case of flexible proteins that may bind ligands in multiple different orientations. As an alternative, end–point methods aim to provide a balance between accuracy and efficiency in Δ*G*_*bind*_ computation, and position themselves between fast docking/scoring approaches and rigorous alchemical strategies for Δ*G*_*bind*_ calculation. They combine explicit conformational sampling (typically in molecular dynamics (MD) simulation) with a relatively fast scoring approach. By definition, end–point methods only require initial and final states to be simulated, i.e., the ligand free in solution and bound to the target protein, respectively, and/or interactions being either turned off and on (Wang et al., 2019).

Linear interaction energy (LIE) is an end–point method that was introduced in 1994 by Åqvist and coworkers (Åqvist et al., 1994). It is derived from the Linear Response Approximation (LRA) (Lee et al., 1992) to compute the electrostatic contributions to the binding affinity. As such, LIE is directly derived from the Zwanzig expression for free–energy perturbation (Leach, 2001). The non-polar contribution to Δ*G*_*bind*_ is in LIE also represented by calculating differences in average non-bonded (i.e., van der Waals) interaction energies between the ligand and its environment in either the protein-bound or unbound state (Åqvist et al., 1994). To compute Δ*G*_*bind*_ from the simulations of the ligand either bound to the protein or free in solvent, the obtained average van der Waals (*vdw*) and electrostatic (*ele*) interaction energies of the ligand with its environment are scaled by LIE parameters α and β:

(1) $$\begin{array}{l} \Delta G_{bind}=\alpha \left({\langle V_{lig-surr}^{vdw}\rangle }_{bound}-{\langle V_{lig-surr}^{vdw}\rangle }_{unbound}\right)\\ +\beta \left({\langle V_{lig-surr}^{ele}\rangle }_{bound}-{\langle V_{lig-surr}^{ele}\rangle }_{unbound}\right) \end{array}$$

Originally LRA was followed and β was set to 0.5 (Åqvist et al., 1994). In subsequent studies (Åqvist and Hansson, 1996; Hansson et al., 1998) Åqvist and co-workers assigned values to β based on electrostatic properties and chemical composition of the compounds of interest. From free energy perturbation studies on the electrostatic contribution to solvation free energies Δ*G*_*solv*_ (Åqvist and Hansson, 1996) and binding affinity prediction for 18 protein-ligand complexes (Hansson et al., 1998) it was concluded that for charged compounds β = 0.5 can be used, while lower values for different types of neutral ligands were found to best describe the electrostatic contribution to Δ*G*_*bind*_ and Δ*G*_*solv*_ (β = 0.43, 0.37, and 0.33 for neutral molecules with 0, 1, or (more than) 2 hydroxyl groups, respectively). The deviation from linear response for neutral compounds and the decrease in assigned β values with the number of hydroxyl groups were explained to originate from variations in solvent reordering around and interactions with the ligands (Åqvist and Hansson, 1996). Accordingly pre-assigned values for β have been used since then in various LIE binding affinity studies; see e.g., Shamsudin Khan et al., 2014 for a recent example in which these values were successfully used, in efforts to automate LIE binding free energy prediction within a drug-design context. Assignment of β based on the chemical nature of the compound of interest has also been extended toward other (hydrogen-bonding) functional groups in a large-scale (solvation) free energy perturbation study by Almlöf et al. (2007). They used a set of hundreds of small organic molecules to derive a model in which β values are assigned based on the number and types of functional groups of the compounds of interest, where each functional group adds a pre-defined perturbation to the base value for β (of 0.43).

We and others (see e.g., Carlson and Jorgensen, 1995; Wall et al., 1999) have chosen to incorporate β as an effective parameter in LIE binding free energy models and (together with α) train it based on experimentally available affinity data. In such cases, separate local models (with different values for α and β) may well be needed to accurately describe binding affinities for complete sets of binders for a given protein of interest, as shown e.g., in van Dijk et al., 2017 for 132 inhibitors of Cytochrome P450 19A1 (CYP19A1). Note that the meaning of empirically calibrated values of the parameters in trained LIE models is not always obvious and/or discussed. One of the exceptions is work of Kollman and co-workers (Wang et al., 1999) who found a correlation between α and the hydrophobicity of the binding site of the system of interest (with a larger number of hydrophobic groups buried after binding resulting in higher affinity and α values). After α and β are pre-assigned and/or calibrated based on experimental data, Equation (1) can be used to predict binding affinities of ligands with unknown experimental data. An optional offset parameter (often denoted as γ) can be added to Equation (1) as a fitting parameter. Fitted values of γ are typically system dependent and have been related to the hydrophobicity of the binding site (Almlöf et al., 2004). Optimal values for an offset parameter in calibrated models may also depend on the compounds of interest, as we illustrated by deriving local LIE models to predict binding affinities for a (diverse) set of 132 CYP19A1 binders, for which inclusion of an offset parameter would have led to different calibrated γ values in the three obtained local models (van Dijk et al., 2017). The use of additional LIE terms and associated scaling parameters has also been proposed such as the introduction of a γ parameter for the scaling and explicit inclusion of a surface-area term (Carlson and Jorgensen, 1995).

From the above, LIE assumes that intramolecular energies, entropic terms, desolvation effects, or other factors contributing to Δ*G*_*bind*_ can be handled and canceled out by fitting and scaling of the model parameters, as it is assumed to correlate linearly with the intermolecular interactions (Åqvist and Marelius, 2001). This scaling and fitting allows for the calculation of “absolute” (direct) values for Δ*G*_*bind*_. For that purpose it can be critical to include and derive an offset γ parameter for the system under consideration (Almlöf et al., 2004). Having direct Δ*G*_*bind*_ values available makes it straightforward to use a Boltzmann–like statistical weighting scheme to include multiple binding poses of ligands combined into a single prediction of Δ*G*_*bind*_ (Stjernschantz and Oostenbrink, 2010). This is relevant for flexible proteins such as Cytochrome P450s that may bind their ligands in different orientations or that may adopt multiple (partial) conformations upon complexation (Stjernschantz et al., 2008). LIE can also handle diverse ligands in the dataset that may involve too large perturbations to be simulated (which may become impractical for alchemical free energy calculations), while simultaneously accounting for the unbound state of the ligand that is not considered by most empirical scoring functions (Brooijmans and Kuntz, 2003). Section 2 summarizes our recent progress in calibrating (statistically–weighted) LIE models for diverse sets of binders of Cytochrome P450s or other flexible and promiscuous proteins.

When fitting parameters in Equation (1) based on experimental data, it should be realized that use of the resulting LIE model(s) asks for the definition of its (their) domain of applicability in order to be able to assess the reliability of predictions for arbitrary query compounds (Carrió et al., 2014). This is especially relevant when using LIE models in industrial or other applied settings, considering e.g., that some years ago the Organisation for Economic Cooperation and Development (OECD) formalized applicability domain (AD) assessment as principle to evaluate model validity (Jaworska et al., 2005). To enable reliability estimation of LIE predictions based on simulation and training data only, we recently introduced strategies to assign the AD of LIE or other protein-structure and -dynamic based models (as reviewed in section 3). Section 4 lists several software tools that have come available to facilitate (semi-)automated LIE modeling. These include our software for automated (statistically–weighted) LIE computation and associated AD assessment, and the availability of these and other tools may well be an important next step for applied use of LIE.

## 2. Statistical Weighting of Multiple Protein-Ligand Binding Conformations

Some years ago, Stjernschantz and Oostenbrink (2010) introduced an extended version of the LIE method in which results from multiple MD simulations starting from different protein conformations and/or binding orientations are combined into a single Δ*G*_*bind*_ calculation. With this method, protein–conformational sampling and the description of ligand–binding promiscuity can be improved when computing Δ*G*_*bind*_ for e.g., Cytochrome P450s or other flexible proteins that may bind their ligand in different binding orientations. The contribution of an individual simulation *i* that starts from a given protein conformation and ligand–docking pose is scaled via a Boltzmann–like statistical weighting scheme as follows (Hritz and Oostenbrink, 2009):

(2) $$\begin{array}{r} W_{i}=\frac{e^{-\Delta G_{bind,i}/k_{B}T}}{\underset{i}{\sum }e^{-\Delta G_{bind,i}/k_{B}T}} \end{array}$$

with Δ*G*_*bind,i*_ the binding free energy calculated from simulation *i* according to Equation (1). The individual weights are then used to calculate Δ*G*_*bind*_ for a compound from *N* different simulations via:

(3) $$\begin{array}{l} \Delta G_{bind}=\alpha \sum_{i}^{N}W_{i}\left({\langle V_{lig-surr}^{vdw}\rangle }_{bound,i}-{\langle V_{lig-surr}^{vdw}\rangle }_{unbound}\right)\\ \;+\beta \sum_{i}^{N}W_{i}\left({\langle V_{lig-surr}^{ele}\rangle }_{bound,i}-{\langle V_{lig-surr}^{ele}\rangle }_{unbound}\right) \end{array}$$

Because the weights *W*_*i*_ are directly dependent on the values of α and β, model calibration based on experimental data has now to be performed using an iterative fitting scheme (Stjernschantz and Oostenbrink, 2010). Hence, this extended version of LIE is also referred to as *iterative* LIE.

This approach was first tested for thiourea binding to Cytochrome P450 2C9 and provided a model with high accuracy when including simulations starting from multiple ligand–binding poses, whereas experimental accuracy could not be obtained when using a single MD simulation per compound (Stjernschantz and Oostenbrink, 2010). Subsequently, model improvement was also shown for thiourea binding to Cytochrome P450 2D6 by using not only different ligand poses but also multiple protein starting structures for MD (Perić–Hassler et al., 2013). The method was further extended by using multiple replicates per docking poses to further increase accuracy (Perić–Hassler et al., 2013). Later, our group has successfully used this Boltzmann–weighting LIE scheme for binding affinity prediction to e.g., CYP1A2 (Capoferri et al., 2015), CYP19A1 (van Dijk et al., 2017), JAK2 kinase (Capoferri et al., 2017), and FXR (Rifai et al., 2018), and it has been implemented in an automatic way in the *eTOX ALLIES* (Capoferri et al., 2017) and *MDStudio* platforms (van Dijk, 2017) (section 4). As part of these efforts, Vosmeer et al. proposed a Fourier–transform filtering strategy to detect stable parts of MD time series of the interaction energy terms (Vosmeer et al., 2016). Only segments with fluctuations smaller than a pre–defined cut–off were subsequently used to average ligand–surrounding interaction energies over. Using previously calculated Δ*G*_*bind*_ data of Cytochrome P450 2D6 (Vosmeer et al., 2014), this filtering strategy was able to make LIE calculation slightly more accurate while potentially greatly improving compute efficiency (Vosmeer et al., 2016). The reason that such filtering does not only improve efficiency but can also enhance accuracy is that the weighting scheme of Equations (2) and (3) is only valid when using results from individual simulations that cover well-separated parts of the potential energy surface of the system of interest (Hritz and Oostenbrink, 2009). Note that Nunes–Alves and Arantes (Nunes–Alves and Arantes, 2014) used a similar Boltzmann–weighting approach to incorporate multiple binding modes into their binding affinity prediction using an implicit solvent model and they tested it on four different receptors.

Besides calculating Δ*G*_*bind*_ with the inclusion of several binding poses in LIE, it was also shown that the probability of a binding pose to occur can be predicted by inspecting the weighting values obtained from Equation (2) (Rifai et al., 2019). We verified this recently for a system of SIRT1–ligands and found for the considered compounds a correlation between the simulations of the protein–bound state with highest weight *W*_*i*_ and information from a co–crystallization study, in terms of the observed protein–ligand interactions and/or the starting poses used in simulation (as compared to the co-crystallized binding poses) (Rifai et al., 2019). Thus, when being able to generate and select appropriate binding poses from docking and/or experimental information on protein–ligand interactions for (a vast majority of the) training compounds, iterative LIE training may well be subsequently performed in an unsupervised manner.

## 3. Applicability Domain Analysis for LIE

Provided that we use the LIE framework as a purely empirical method, i.e., not considering categories of the β parameter based on the chemical nature of the ligand (Hansson et al., 1998; Almlöf et al., 2007), the need for training and the use of fitted parameters (α and β) may raise the question of how reliable the predicted value will be for an arbitrary query compound. Hence there is a need to provide a measure to estimate the reliability of a prediction for a new compound with unknown binding affinity, and to evaluate if the query compound of interest is sufficiently represented by the employed set of model training compounds. This can be expressed in terms of the *applicability domain* (AD) of a given LIE model. The AD is a set of knowledge or information on the training set of the model and can give a measure for the confidence in a given prediction, in a similar vein as commonly applied in ligand–based empirical approaches (Carrió et al., 2014). A few years ago our group introduced an approach to allow AD assignment of LIE models, based on simulation and training data only (Capoferri et al., 2015, 2017; van Dijk et al., 2017). To our knowledge, this is the first method to analyze the AD of protein–structure (and –dynamic) based models such as LIE.

Inspired by a previous applicability domain analysis (ADAN) approach of Pastor and co–workers to define the domain of applicability of ligand–based QSAR models (Carrió et al., 2014), we have proposed an AD analysis strategy in a LIE study on Cytochrome P450 1A2 binding (Capoferri et al., 2015). In this study a relatively large set of (57) structurally–diverse training and test compounds were employed to explore the possibility to define the AD of calibrated LIE models in terms of five metrics. To estimate the reliability of a given prediction, these metrics or *confidence indices* are used to evaluate the similarity of an arbitrary query ligand (for which Δ*G*_*bind*_ is to be predicted) with the model's training set. This is not only evaluated in terms of structural similarity (according to Tanimoto scores) and computed Δ*G*_*bind*_ (as compared to the spread in experimental data used for calibration), but also in terms of the characteristics of the protein–ligand interactions. For the latter purpose, Mahalanobis–distance and (two) principal–component analyses are performed to enable a quantitative comparison between the averaged and the most relevant per–residue van der Waals and electrostatic interactions during simulation of either the protein–bound query or training compounds (Vosmeer et al., 2014; Capoferri et al., 2015). With these metrics in hand and after splitting the set of ligands with known binding affinity into a training and test set (of 35 and 22 compounds, respectively), a distinction could be successfully made between accurate and inaccurate Δ*G*_*bind*_ predictions for the test set compounds by looking at how many of the confidence metrics were violated per prediction (Capoferri et al., 2015).

An important conclusion from Capoferri's AD analysis was that the nature of the protein–ligand interactions (in terms of averaged non-bonded energies and the involved interacting protein residues) were more relevant descriptors for the AD of the LIE model than the molecular structure of the ligands alone. In the LIE study of van Dijk et al. mentioned in section 1 (van Dijk et al., 2017), this finding was confirmed for local models that were inferred for a set of 132 structurally–diverse CYP19A1 binders. By profiling and comparing per–residue interactions as observed for the protein–ligand simulations used for training, van Dijk showed differences in protein–ligand interactions among the three local models inferred, while structurally related compounds were not necessarily part of the same local model, indicating that protein–ligand interactions are a better measure to quantify if a given compound falls within the AD of a LIE model when compared to the molecular structure or other properties of the ligand alone.

The performances of LIE and the above mentioned AD analysis approach were evaluated in a real–life scenario of a community blind affinity prediction challenge organized by Drug Design Data Resource (D3R) during phase 2 of Grand Challenge 2 (GC2) (Gaieb et al., 2018). In D3R GC2, the challenge was to predict binding affinities of (102) agonists with different scaffolds for nuclear receptor FXR. For a subset of benzimidazole compounds (*n* = 9), a predictive accuracy (with a deviation from experiment of less than 5 kJ mol^−1^) was obtained. Importantly, we showed that our AD analysis can yield representative metrics (in terms of an index for the level of confidence) to quantify the reliability of the binding affinity predictions based on simulation data only. It should also be noted that LIE might fail to predict the binding affinity of compounds with different structural properties and protein–ligand interaction profiles and/or domain of applicability from the ligands used for model training, or when the number of compounds constituting the training set cannot cover the range of experimental data of the test set. However, this can be estimated by the confidence level retrieved from AD analysis, to indicate possible limitations of the obtained LIE model. To enrich the interpretation of the applicability domain, we incorporated protein–ligand interaction profiling to evaluate the interaction of FXR with its ligands per obtained confidence level. We found that the confidence levels of the AD analysis were in line with the frequencies of ligand interactions with hotspot residues in the protein and with the model deviation and correlation metrics obtained from the predictions (Rifai et al., 2018).

## 4. (Semi-)Automated LIE Modeling and Analysis Tools

Several software modules or packages (Table 1) are available that can be used to facilitate LIE modeling, such as the built–in package *gmx lie* within GROMACS (van der Spoel et al., 2005; Abraham et al., 2015) which can be used to directly obtain free energies of binding from interaction energy term analyses. *Q* (Marelius et al., 1998; Bauer et al., 2018) can semi–automatically perform LIE and FEP calculations, and is assisted with a Graphical User Interface (GUI) (Isaksen et al., 2015). The *Free Energy Workflow (FEW)* tool (Homeyer and Gohlke, 2013, 2015) also enables LIE (as well as other free energy) calculations by facilitating setup and execution within the Amber suite (Case et al., 2005; Salomon–Ferrer et al., 2013). *CaFE* (Liu and Hou, 2016) specializes in calculating Δ*G*_*bind*_ by using end–point methods including LIE. *FESetup* (Loeffler et al., 2015) can facilitate alchemical free energy simulations and provides the ability to perform end-point calculations as well. *Desmond* (Bowers et al., 2006; Gao et al., 2012) from Schrödinger can also be used to extract ligand–surrounding interaction energies from MD simulations. Our *eTOX ALLIES* pipeline (Capoferri et al., 2017) enables automated molecular docking, MD simulation, iterative LIE and associated AD analysis, which can be based on inclusion of multiple binding modes and/or protein conformations as input for the MD simulations (Capoferri et al., 2017). Recently, we have made such LIE workflow also available within our modular and flexible *MDStudio* workflow management system (van Dijk, 2017).

**Table 1:** Selection of available tools to perform, facilitate and/or automate LIE calculations.

**Software**	**Operating system**	**Free/Commercial**	**Requirement**	**Type**
*gmx lie*	Windows, Linux, MacOS	Free	GROMACS	Program in simulation software
*Q*	Windows, Linux, MacOS	Free	–	Simulation software
*FEW*	Windows, Linux, MacOS	Free^*^	Amber, AmberTools	Perl script
*CaFE*	Windows, Linux, MacOS	Free	VMD	Tcl scripts
*eTOX ALLIES*	Windows, Linux, MacOS	Free	– (all required softwares are in the virtual machine)	Python scripts in virtual-machine environment
*MDStudio*	Windows, Linux, MacOS	Free^*^	Open Babel, PLANTS, AmberTools and GROMACS	Python scripts in docker and microservice environment
*FESetup*	Windows, Linux, MacOS	Free^*^	GROMACS, Amber, Sire, NAMD	Shell script
*Desmond*	Windows, Linux, MacOS	Commercial	Schrödinger suite	Software

* *These tools (may) require Amber or PLANTS, which are commercial softwares*.

## 5. Conclusions

The current perspective summarizes how we have explored the use of (statistically-weighted) LIE to predict binding affinity for challenging flexible (off-)target proteins such as Cytochrome P450s and nuclear receptor FXR. In addition we reviewed possibilities to evaluate the confidence in LIE predictions with an AD assessment approach for LIE or other protein-structure and -dynamic based free energy methods. Especially when the AD of a LIE model can be defined, LIE can treat sets of ligands that may involve too large perturbations to be simulated and become impractical for alchemical free energy perturbation or thermodynamic integration, while simultaneously accounting for the unbound state of the ligand that is not considered by most combined docking/scoring approaches. Thus, calibrated LIE models can be viewed as a combination of (4D–)QSAR and sampling approaches to estimate protein–binding affinities. Combined with the possibility to employ tools that facilitate and automate LIE calculations and (AD) analysis, the potential of addressing protein flexibility and promiscuity in statistically-weighted models and the availability of metrics for applicability domain analysis show direct promises for use of LIE in applied settings.

## Author Contributions

ER, MD, and DG contributed to drafting, writing and editing of the manuscript.

## Conflict of Interest

The authors declare that the research was conducted in the absence of any commercial or financial relationships that could be construed as a potential conflict of interest.
